# Clinical Efficacy of Immune Checkpoint Inhibitors in Older Non-small-Cell Lung Cancer Patients: A Meta-Analysis

**DOI:** 10.3389/fonc.2020.558454

**Published:** 2020-09-23

**Authors:** You-Meng Sun, Ying Wang, Xin-Xing Sun, Jing Chen, Zhi-Ping Gong, Hai-Yan Meng

**Affiliations:** The First Affiliated Hospital, Zhejiang University School of Medicine, Hangzhou, China

**Keywords:** immune checkpoint inhibitors, PD-1/PD-L1, chemotherapy, non-small- cell lung cancer, meta-analysis

## Abstract

**Background:** Immune checkpoint inhibitors (ICIs) have transformed the treatment landscape among non-small-cell lung cancer (NSCLC) patients. The efficacy of ICI therapy in older patients (≥65 years) is controversial and not fully clarified. We performed a systematic review and meta-analysis to evaluate the efficacy of ICIs in patients with advanced or metastatic NSCLC based on age (<65 years vs. ≥65 years).

**Methods:** A comprehensive literature search for eligible randomized control phase II/III trials that compared the efficacy of anti-PD-1/PD-L1 agents against chemotherapy in advanced or metastatic NSCLC patients. Pooled overall survival (OS) and progression-free survival (PFS) estimates were calculated based on random/fixed effects models according to the heterogeneity between the studies.

**Results:** A total of 10 studies involving 8 randomized controlled trials (2 updates) were enrolled in this meta-analysis [2,662 young patients (<65 years) and 1,971 older patients (≥65 years)]. The efficacy of anti-PD-1/PD-L1 agents is comparable between young (<65 years) and older (≥65 years) patients for OS [HR 0.75 95% CI (0.64–0.88) vs. 0.76 95% CI (0.66–0.87)]. However, our pooled analysis was not sufficient to show a significant benefit in terms of PFS for anti-PD-1/PD-L1 agents [HR 0.87 95% CI (0.74–1.01), *P* = 0.06]. In addition, we failed to see a PFS superiority of anti-PD-1/PD-L1 agents against chemotherapy in two age subgroups [<65 years and ≥65 years, HR 0.85 95% CI (0.72–1.01), *P* = 0.07 and HR 0.87 95% CI (0.68–1.10), *P* = 0.25].

**Conclusion:** ICIs therapy presents comparable efficacy in older advanced or metastatic NSCLC patients with young patients.

## Introduction

Nowadays lung cancer remains to be the leading cause of cancer-related death all over the world. Non-small-cell lung cancer (NSCLC) accounts for more than 80% of newly diagnosed lung cancer (1). In addition, patients diagnosed with lung cancer are typically older and the median age is 70 years old (2). Advanced NSCLC treatment has achieved great progress with the introduction of immune checkpoint inhibitors (ICIs) including cytotoxic T-lymphocyte-associated antigen-4 (CTLA-4), programmed cell death protein-1 (PD-1) and its ligand programmed death receptor ligand-1 (PD-L1). Several ICIs with promising efficacy have been approved for the treatment of NSCLC, including Nivolumab (3, 4), Pembrolizumab (5), Atezolizumab (6), and Durvalumab (7). However, elderly patients are generally underrepresented for most ICIs clinical trials involve low proportion of elderly patients as a result of multiple comorbidities and decline in organ function (8). Recent evidence about benefits from ICIs between young and elder patients is controversial. A meta-analysis including 9 randomized controlled trials (5 comprising NSCLC patients) reported similar overall survival (OS) and progression-free survival (PFS) between younger (<65 years) and older (≥65 years) patients (9). Wu et al. found older (≥65 years) patient derived better benefits than younger (<65 years) patients from the use of ICIs (10). However, patients more than 80 years old were reported to have shorter PFS compared with other age groups (11).

Clinical efficacy of ICIs in elderly NSCLC patients has not been fully assessed. In order to address this question, we performed a systematic review and meta-analysis of randomized controlled trials to evaluate the efficacy of ICIs based on age.

## Methods

### Study Eligibility and Identification

A systematic literature search of PubMed, Embase, Cochrane Library and Clinical trials was performed to identify eligible RCTs that compared Food and Drug Administration (FDA) approved anti-PD-1/PD-L1 agents as first-line therapy against chemotherapy in patients with advanced or metastatic NSCLC from inception to April 2020. The language was limited to English. The following Medical Subject Headings (MeSH) terms and related variants were used: “Carcinoma, non-small-cell lung,” “NSCLC,” “Nivolumab,” “Pembrolizumab,” “Atezolizumab,” “Avelumab,” “Durvalumab,” “Cemiplimab,” “randomized controlled trial.” Moreover, we manually looked into relevant references of systematic reviews, meta-analyses to search for additional studies. Additionally, the American Society of Clinical Oncology, the European Society of Medical Oncology, and the International Association for the Study of Lung Cancer were searched for relevant new evidence. The comprehensive PubMed search strategy was provided in Supplementary Table 1. The inclusion criteria were (1) Phase II/III randomized controlled trials compared the survival of single agent PD-1/PD-L1 inhibitors with chemotherapy in patients with advanced or metastatic NSCLC. (2) Reported the hazard ratio (HR) for overall survival (OS) and/or progression-free survival (PFS) based on stratification of age (<65 years and ≥65 years). The exclusion criteria were (1) Reviews, meta-analysis or pooled analysis, case report, guidelines and expert consensus, single-arm trial. (2) Combined therapy (e.g., Pembrolizumab plus chemotherapy vs. chemotherapy).

### Data Extraction and Quality Assessment

The following information was extracted from eligible studies by two investigators independently: first author, year of publication, study name, National Clinical Trial (NCT) number, trial phase, study arms, the number of patients in total and age subgroups(<65 years and ≥65 years), HR for OS and PFS, HR for OS and/or PFS based on age subgroups (<65 years and ≥65 years). Two investigators independently assessed the quality of the RCTs by using Cochrane risk assessment tool, and resolved the discrepancies through discussion and consult with a third one.

### Outcome Measures

The primary outcomes were HR for overall survival and progression-free survival, OS defined as time from randomization to death from any cause, PFS defined as time from randomization until the first occurrence of disease progressive according to Response Evaluation Criteria in Solid Tumors (RECIST) version 1.1 or death from any cause, the secondary outcome were HR for OS and/or PFS based on stratification of age (<65 years and ≥65 years).

### Statistical Analyses

Pooled OS and PFS estimates were calculated based on random/fixed effects models according to the heterogeneity between the studies. Cochran's Q test was used to assess heterogeneity between the studies and *I*^2^ was calculated to evaluate the degree of inconsistency. Combined estimates (≥ 65 years) was developed with random effects model for the studies that reported separate HR estimates for 65–75 and >75 years. Statistical analyses were performed using the metafor package in R, version 3.2.3 (R foundation for statistical computing). The *P* < 0.05 was deemed to be statistically significant.

## Results

### Study Selection and Patient Characteristics

**Figure 1:**
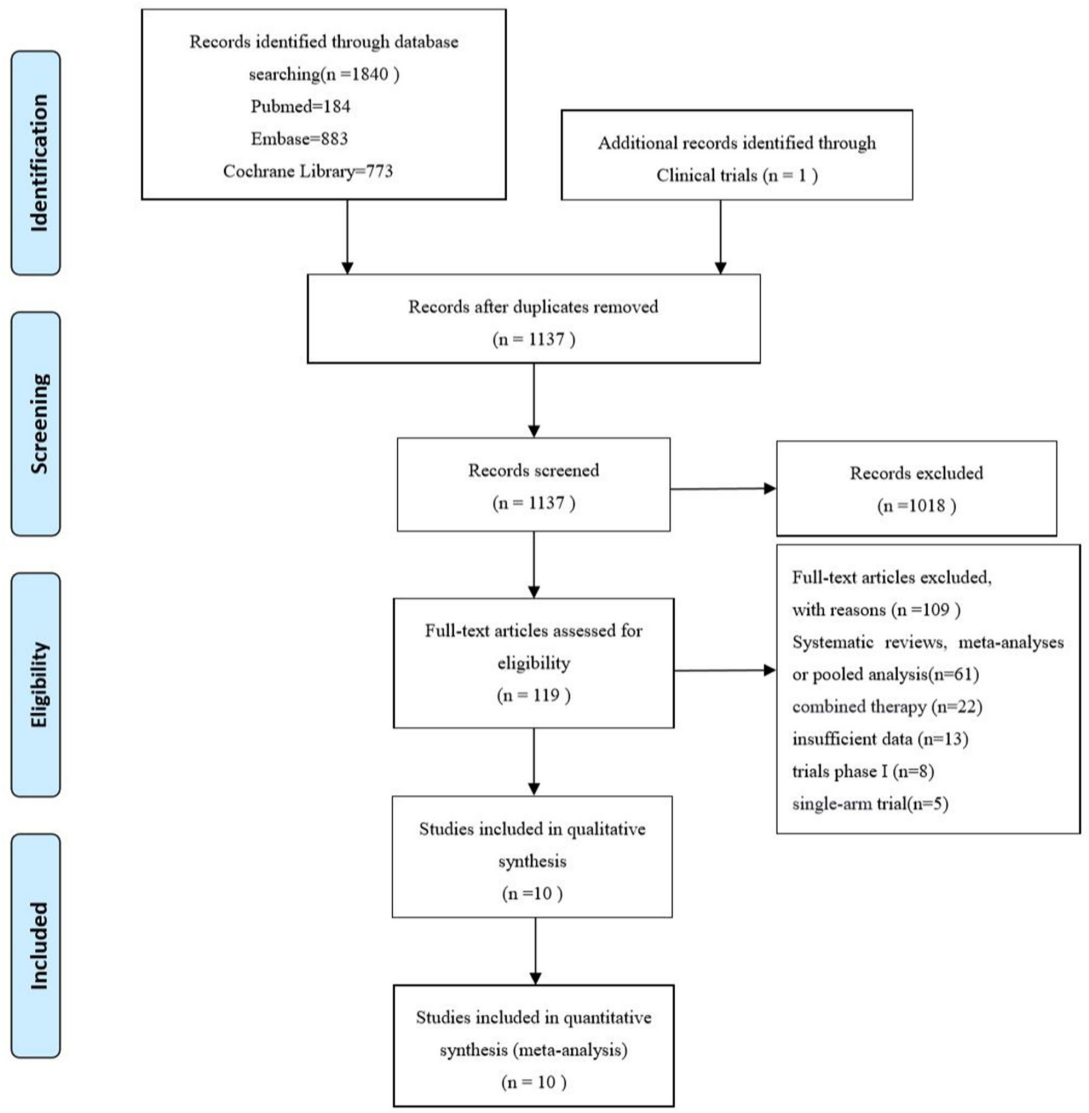
PRISMA flow diagram of the studies eligible for meta-analysis.

A total of 1,840 records were initially retrieved from PubMed, Embase, Cochrane Library and 1 additional record identified through Clinical trials up to April 2020. Among them, 1,137 records were kept after duplicates, and 1,018 records were removed by screening the title and abstract. After full-text reading, 109 records were excluded, systematic reviews, meta-analysis or pooled analysis (*n* = 61), combined therapy with other agents (*n* = 22), insufficient data based on age subgroup (*n* = 13) trials phase I (*n* = 8), single-arm trial (*n* = 5). Finally, 10 studies (3–6, 12–17) including 8 randomized controlled trials and 2 updates (14, 17) were incorporated in this meta-analysis. The PRISMA flow diagram of study selection is shown as follows (Figure 1). A total of 4,633 patients including 2,662 young patients (<65 years) and 1,971 older patients (≥65 years) with advanced or metastatic NSCLC were enrolled. Among the 8 RCTs, 3 trials (3, 4, 15) investigated Nivolumab (anti-PD-1 agents), 2 trials (5, 13, 14) investigated Pembrolizumab (anti-PD-1 agents), 2 trials (6, 12, 17) investigated Atezolizumab (anti-PD-L1 agents), 1 trial (16) investigated Avelumab (anti-PD-L1 agents); 1 (12, 17) trial phase 2 study, 1 (5) trial phase 2/3 study, 6 (3, 4, 6, 13–16) trials phase 3 studies; 6 trials (3–6, 12, 16, 17) compared PD-1/PD-L1 inhibitors monotherapy with docetaxel and 2 trials (13–15) compared PD-1/PD-L1 inhibitors monotherapy with platinum-based chemotherapy. The characteristics of the included trials are detailed in Table 1 and the quality assessment is presented in Supplementary Figure 1.

**Table 1:** Characteristics of Included Trials.

**References**	**Study name**	**NCT number**	**Drug**	**Phase**	**Malignancy**	**Arm1**	**Arm2**	**Patient' number**	***n* < 65 y**	***n* ≥ 65 y**
Brahmer et al. (3)	CheckMate017	NCT01642004	Nivolumab	3	S-NSCLC	Nivolumab 3 mg/kg every 2 weeks	Docetaxel 75 mg/m^2^ every 3 weeks	272	152	120
Borghaei et al. (4)	CheckMate057	NCT01673867	Nivolumab	3	NS-NSCLC	Nivolumab 3 mg/kg every 2 weeks	Docetaxel 75 mg/m^2^ every 3 weeks	582	339	243
Fehrenbacher et al. (12, 17)	POPLAR	NCT01903993	Atezolizumab	2	NSCLC	Atezolizumab 1,200 mg every 3 weeks	Docetaxel 75 mg/m^2^ every 3 weeks	287	169	118
Herbst et al. (5)	KEYNOTE-010	NCT01905657	Pembrolizumab	2/3	NSCLC	Pembrolizumab 2 mg/kg every 3 weeks Pembrolizumab 10 mg/kg every 3 weeks	Docetaxel 75 mg/m^2^ every 3 weeks	1,033	604	429
Reck et al. (13) & Reck et al. (14)	KETNOTE-024	NCT02142738	Pembrolizumab	3	NSCLC	Pembrolizumab 200 mg every 3 weeks	Investigator's choice of platinum-based chemotherapy	305	141	164
Rittmeyer et al. (6)	OAK	NCT02008227	Atezolizumab	3	NSCLC	Atezolizumab 1,200 mg every 3 weeks	Docetaxel 75 mg/m^2^ every 3 weeks	850	453	397
Carbone et al. (15)	CheckMate026	NCT02041533	Nivolumab	3	NSCLC	Nivolumab 3 mg/kg every 2 weeks	Investigator's choice of platinum-based chemotherapy	271	148	123
Barlesi et al. (16)	JAVELIN lung 200	NCT02395172	Avelumab	3	NSCLC	Avelumab 10 mg/kg every 2 weeks	Docetaxel 75 mg/m^2^ every 3 weeks	529	279	250

*NSCLC, non-small-cell lung cancer; S-NSCLC, squamous non-small-cell lung cancer; NS-NSCLC, non-squamous non-small-cell lung cancer; NCT Number, National Clinical Trial number*.

### Overall Survival

**Figure 2:**
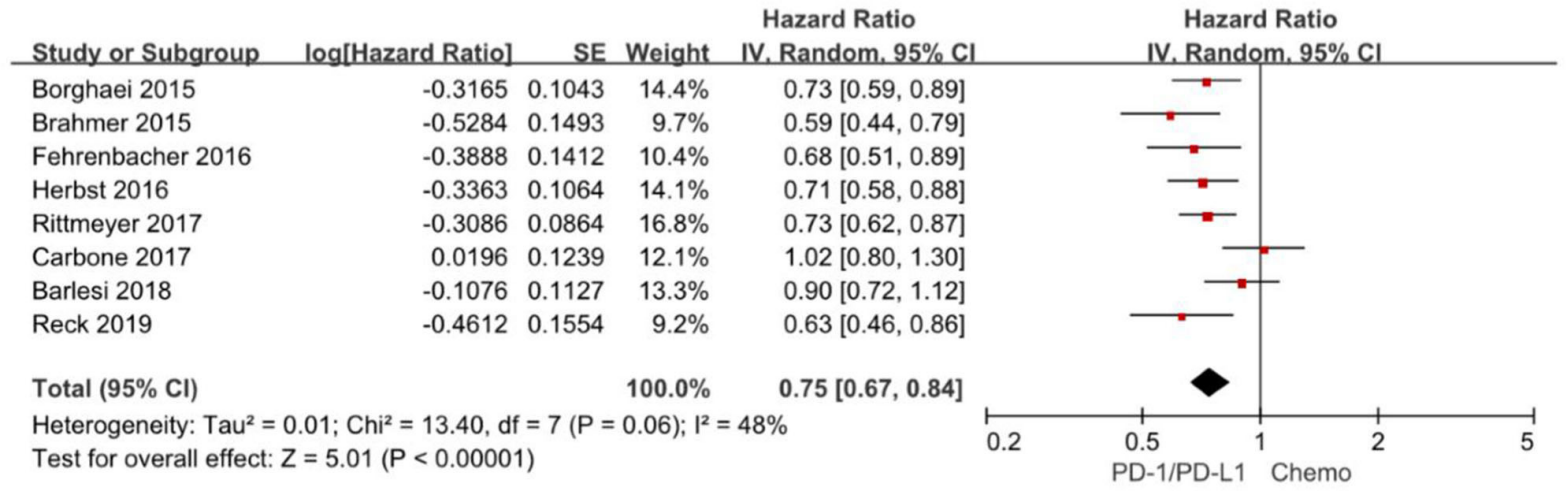
Forest plot for OS. Studies are listed on the left and HR with 95% CI are on the right. Box sizes are inversely proportional to the standard error of the study; therefore, larger boxes indicate greater weight of the trial in the meta-analysis estimation.

Overall survival is often considered as gold standard and the most clinically relevant primary outcome in clinical trials. The hazard ratios of individual studies and the combined results were illustrated in Figure 2 and the results presented the efficacy of anti-PD-1/PD-L1 agents against chemotherapy according to overall survival. The pooled HR of overall survival based on random-effects models is 0.75 with 95% CI of 0.67–0.84 (*P* < 0.00001), which implies anti-PD-1/PD-L1 agents with a 25% reduction in the risk of death compared to chemotherapy in the overall population.

The chi-square test for heterogeneity was not significant (*P* = 0.06), indicating no substantial difference between the individual trials results.

### Overall Survival Based on Age Subgroup

**Figure 3:**
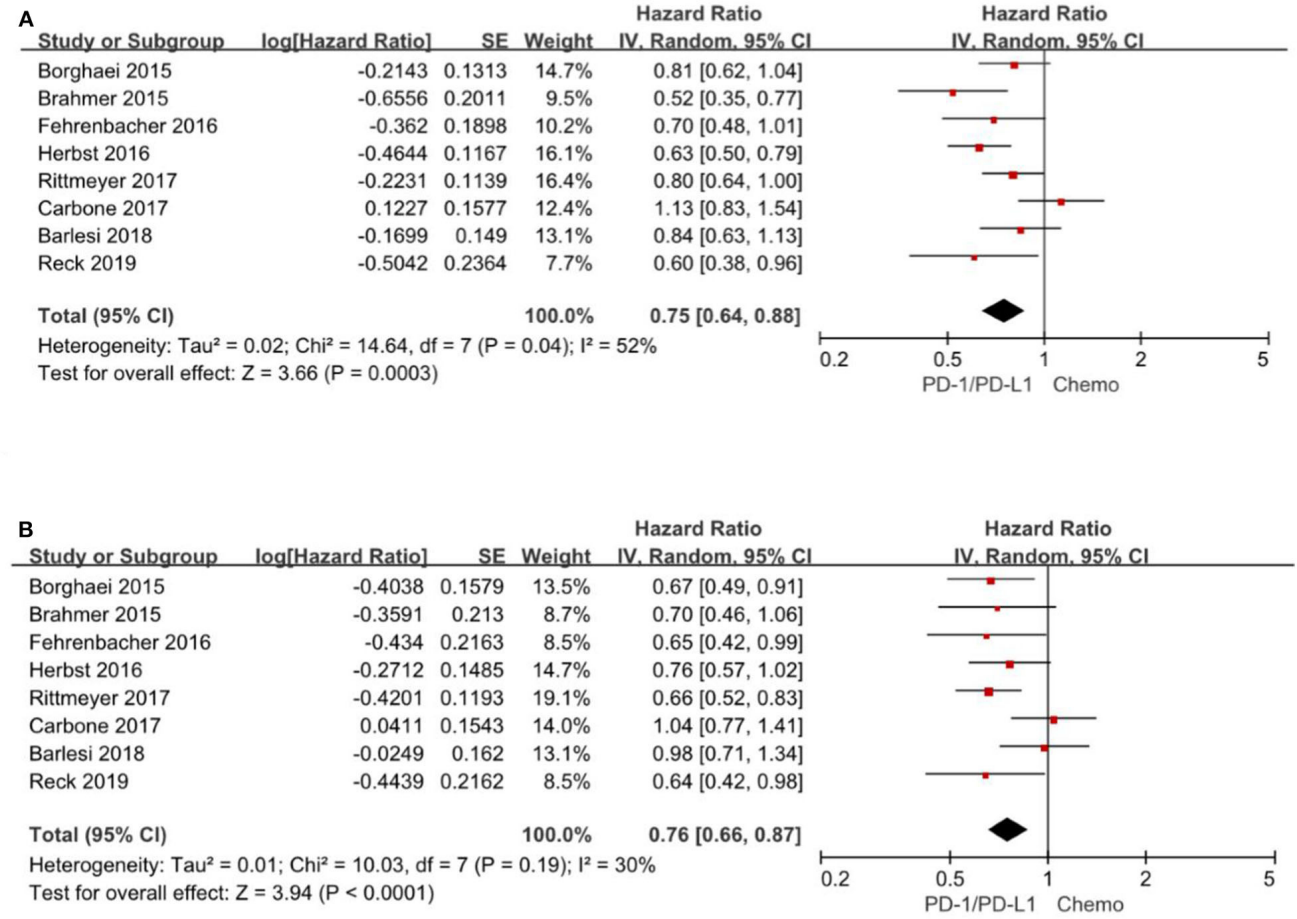
Forest plot for OS for patients <65 years **(A)** and ≥65 years **(B)**. Studies are listed on the left and HR with 95% CI are on the right. Box sizes are inversely proportional to the standard error of the study; therefore, larger boxes indicate greater weight of the trial in the meta-analysis estimation.

All the 8 trials (3–6, 14–17) reported the HR for overall survival based on age subgroup, among them 1,971 (42.5%) patients were older than 65 years and age ranged from 21 to 90 years. For patients <65 years, the pooled HR is 0.75 with 95% CI of 0.64–0.88 (*P* = 0. 0003). There is evidence of differences (*P* = 0.04), indicating considerable inconsistency between the individual trials results. For patients ≥65 years, the pooled HR is 0.76 with 95% CI of 0.66–0.87 (*P* < 0.0001) and there exist no differences between the individual trials studies (*P* = 0.19) (Figure 3). Consequently, the comparable hazard ratios between the two subgroups (<65 years vs. ≥65 years) and overlap of the confidence intervals presented evidence that age have limited effect on overall survival.

### Progression-Free Survival

**Figure 4:**
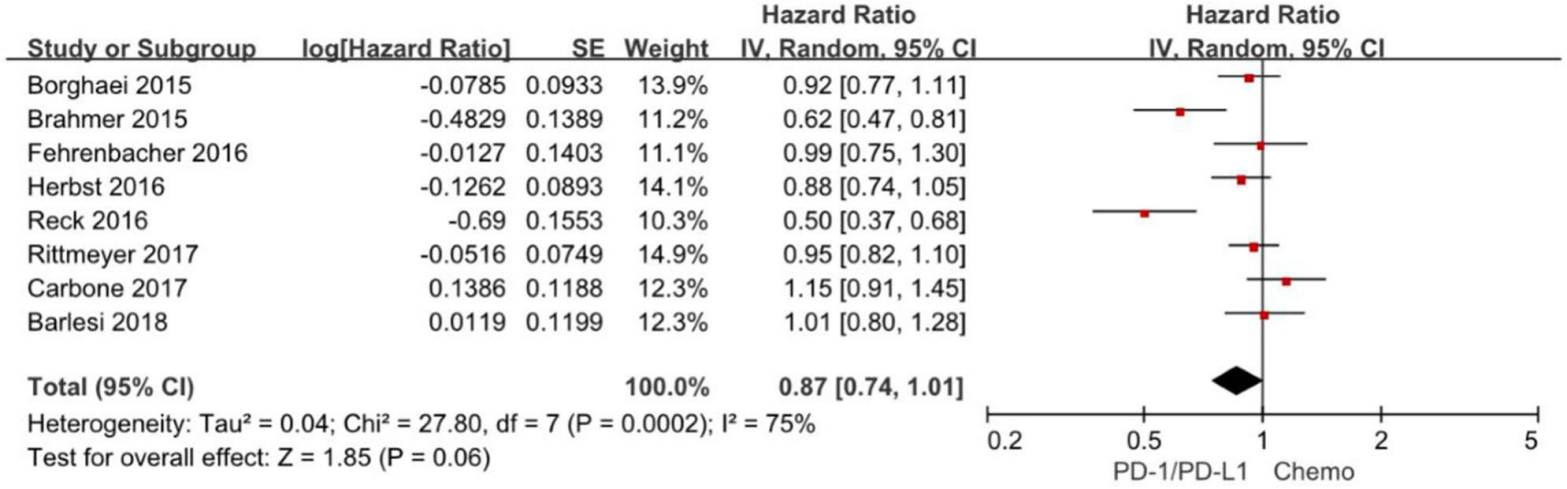
Forest plot for PFS. Studies are listed on the left and HR with 95% CI are on the right. Box sizes are inversely proportional to the standard error of the study; therefore, larger boxes indicate greater weight of the trial in the meta-analysis estimation.

Progression-free survival is increasingly applied as an important endpoint in oncology clinical trials. The hazard ratios of individual studies and the combined results were illustrated in Figure 4 and the results presented the efficacy of anti-PD-1/PD-L1 agents against chemotherapy according to progression-free survival. The pooled HR of progression-free survival based on random-effects models is 0.87 with 95% CI of 0.74–1.01 (*P* = 0. 06), indicating no evidence of significant efficacy difference between anti-PD-1/PD-L1 agents and chemotherapy in the overall population.

The chi-square test for heterogeneity was highly significant (*P* = 0.0002), indicating substantial difference between the individual trials results.

### Progression-Free Survival Based on Age Subgroup

**Figure 5:**
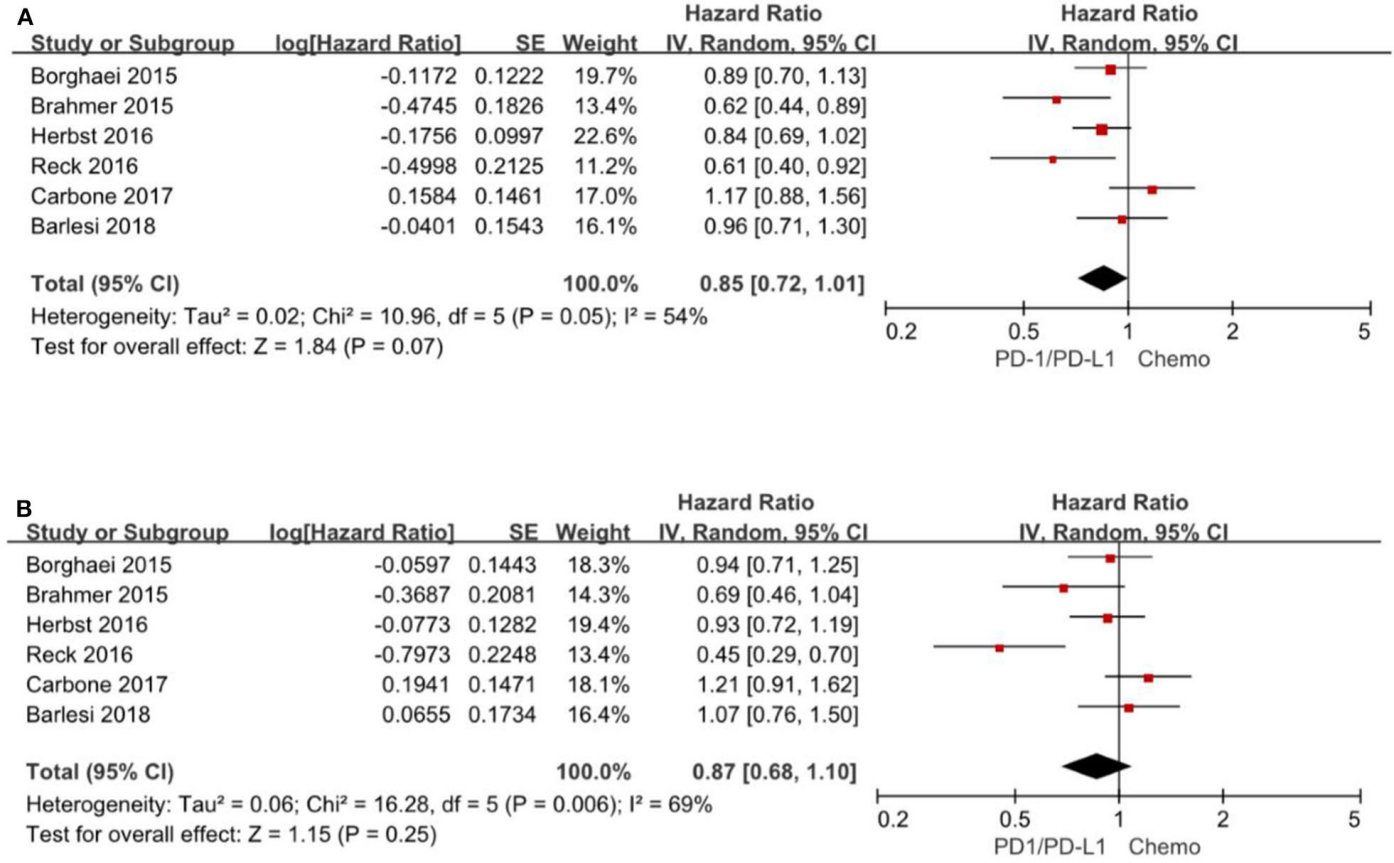
Forest plot for PFS for patients <65 years **(A)** and ≥65 years **(B)**. Studies are listed on the left and HR with 95% CI are on the right. Box sizes are inversely proportional to the standard error of the study; therefore, larger boxes indicate greater weight of the trial in the meta-analysis estimation.

A total of 6 trials (3–5, 13, 15, 16) reported HR for progression-free survival based on age subgroup, among them 1,329 (38.0%) patients were older than 65 years and age ranged from 21 to 90 years. For patients <65 years, the pooled HR is 0.85 with 95% CI of 0.72–1.01 (*P* = 0.07). There is no evidence of heterogeneity (*P* = 0.05). For patients≥65 years, the pooled HR is 0.87 with 95% CI of 0.68–1.10 (*P* = 0.25) and there exist significant differences between the individual trials studies (*P* = 0.006) (Figure 5).

## Discussion

Immunosenescence, defined as changes in the host immunity associated with increased age, accounts for high prevalence of malignancies in elderly people and may influence the efficacy and the activity of ICIs (18). Declined proliferation of T cells is often seen as hallmark of cellular senescence (19). The CD8+ naive T cells, which are the principal elements involved in the PD-1/PDL-1 pathway, will decreases along with aging (20). The co-stimulatory molecule CD28, plays a crucial role in sustaining T cell activation (21). Higher CD8 + CD28- T-cells in older adults (22) leads to impaired immune activation (23, 24) and increase in cancer (25). In addition, blockade of the PD-1 on the surface of T cells is not likely to be efficient to restore T cell activity to the same level of the younger (26).

In this study we found that the efficacy of anti-PD-1/PD-L1 agents is comparable between young (<65 years) and older (≥65 years) patients for OS [HR 0.75 (CI 0.64–0.88) vs. 0.76 (CI 0.66–0.87)]. However, our pooled data were not sufficient to show a significant benefit in terms of PFS for anti-PD-1/PD-L1 agents [HR 0.87 (CI 0.74–1.01), *P* = 0.06]. In addition, we did not see a PFS superiority of anti-PD-1/PD-L1 agents against chemotherapy in two age subgroups [<65 years and ≥65 years, HR 0.85 (CI 0.72–1.01), *P* = 0.07 and HR 0.87 (CI 0.68–1.10), *P* = 0.25]. Our study demonstrated prolonged OS and comparable benefit in patients of age <65 and ≥65 years, which is consistent with a previous meta-analysis (10). Notably, with more randomized controlled trials enrolled in this study (6 vs. 8 studies), age related difference seemed to be reduced [(0.73 vs. 0.69) vs. (0.75 vs. 0.76)], which may attribute to the heterogeneity of the newly enrolled studies with different ICIs agents (Avelumab) and other confounders. In addition, we did not observe prolonged PFS, no matter in young (<65 years) or older (≥65 years) patients. It can be partially explained by the low proportion of elderly patients enrolled and missing data about progression-free survival by age in quite a considerable number of RCTs, which may result in the underestimation of statistically significant difference.

There is still not enough attention paid on the impact of aging on the effectiveness of ICIs for plenty of clinical trials not containing age subgroups. Some studies reporting age subgroups enrolled low proportion of elderly patients that were not proportionate to the real incidence rate of elderly patients in the overall population.

Our meta-analysis has some strengths. We comprehensively collected pooled data of the most up-to-date high-quality randomized controlled trials and provided best level of evidence presenting the efficacy of ICIs in young (<65 years) and older (≥65 years) advanced NSCLC patients. The study enrolled all ICIs that have been applied in the treatment of NSCLC, including two PD-1 inhibitors (Nivolumab, Pembrolizumab) and two PD-L1 inhibitors (Atezolizumab, Avelumab).

Several limitations of the present analysis should be acknowledged. First, without access to original data of each individual patient, we are unable to present more accurate age-dependent outcomes on the efficacy of ICIs therapy. Notably, safety is more important than efficacy when we evaluate a new drug or treatment. Only three studies provided the required data of toxicity events for analysis, otherwise we may provide a more comprehensive knowledge of the safety and efficacy of ICIs in advanced/metastatic NSCLC based on age. Secondly, there exists quite considerable substantial heterogeneity between the included studies, which comes from different ICIs agents, chemotherapy components, PD-L1 expression levels, cancer histotype, age distribution and other relevant factors. A recent meta-analysis suggested PD-1 inhibitors exhibited a better survival outcomes than PD-L1 inhibitors (27), which implied the efficacy difference between PD-1 inhibitors and PD-L1 inhibitors. As to the control intervention, some studies chose docetaxel while the others adopted platinum-based chemotherapy. Plenty of studies have suggested that ICIs therapy benefit correlates with the extent of PD-L1 expression (28–30). Therefore, random-effect model was applied to minimize the influence of these factors. Last but not least, for the underrepresentation of elderly patients in most clinical trials, more large-scale and high-quality randomized controlled trials are required to further confirm the conclusion. What is worth mentioning, most of the clinical trials in the study had enrolled patients with ECOG performance score 0-1. The elderly patients with good performance score could only represent a minority in clinical practice, which may overestimate the efficacy of ICI therapy in older patients. In addition, more real-world studies in older patients would help us better assess the real efficacy and safety of ICIs therapy.

In conclusion, ICIs monotherapy presents survival improvement for both young and older advanced NSCLC patients compared with chemotherapy. The magnitude of improvement would not vary by age. It provides solid evidence that older patients could get the comparable efficacy with young patients, despite of the existence of immunosenescence. Future studied should focus on better strategies to provide precision therapy for elderly patients, including identifying predictive biomarker that accurately reflects ICIs efficacy and developing a comprehensive model of geriatric assessment.

## Core Tip

Our meta-analysis has some strengths. We comprehensively collected pooled data of the most up-to-date high-quality randomized controlled trials and provided best level of evidence presenting the efficacy of ICIs in young (<65 years) and older (≥65 years) advanced NSCLC patients.

## Prisma 2009 Checklist Statement

All authors have read the PRISMA 2009 Checklist, and the manuscript was prepared and revised according to the recommendations of the PRISMA document.

## Data Availability Statement

All datasets generated for this study are included in the article/Supplementary Material.

## Author Contributions

Y-MS and YW finished the initial design and conception of the research, participated in drafting, and revising the article. Y-MS, X-XS, JC, Z-PG, and H-YM contributed to the acquisition of data, analysis, and interpretation. All authors approved the final draft of the manuscript.

## Conflict of Interest

The authors declare that the research was conducted in the absence of any commercial or financial relationships that could be construed as a potential conflict of interest.

## Abbreviations

ICIs: Immune checkpoint inhibitors
PD-1: programmed cell death protein-1
PD-L1: programmed death receptor ligand-1
CTLA-4: T-lymphocyte-associated antigen-4
NSCLC: non-small-cell lung cancer
S-NSCLC: squamous non-small-cell lung cancer
NS-NSCLC: non-squamous non-small-cell lung cancer
OS: overall survival
PFS: progression-free survival
HR: hazard ratio
CI: confidence interval
RCT: randomized controlled trial
NCT: National Clinical Trial
FDA: Food and Drug Administration
MeSH: Medical Subject Headings
RECIST: Response Evaluation Criteria in Solid Tumors.
