# Supplementary material for: Clinical Efficacy of Immune Checkpoint Inhibitors in Older Non-small-Cell Lung Cancer Patients: A Meta-Analysis
Source: Front Oncol. 2020 Sep 23;10:558454. doi: 10.3389/fonc.2020.558454 (PMC7538790; doi:10.3389/fonc.2020.558454)
Supplement: Supplementary file 1 [file Table_1.DOCX]

| **Supplementary TABLE 1 PubMed Search Strategy** | | |
| --- | --- | --- |
| **Step** | **Query** | **Items found** |
| #1: | Search "Carcinoma, Non-Small-Cell Lung"[Mesh] | 51767 |
| #2: | Search (((((((((((Carcinoma, Non Small Cell Lung[Title/Abstract]) OR Carcinomas, Non-Small-Cell Lung[Title/Abstract]) OR Carcinomas, Non-Small-Cell Lung[Title/Abstract]) OR Lung Carcinoma, Non-Small-Cell[Title/Abstract]) OR Lung Carcinomas, Non-Small-Cell[Title/Abstract]) OR Non-Small-Cell Lung Carcinomas[Title/Abstract]) OR Nonsmall Cell Lung Cancer[Title/Abstract]) OR Non-Small-Cell Lung Carcinoma[Title/Abstract]) OR Non Small Cell Lung Carcinoma[Title/Abstract]) OR Carcinoma, Non-Small Cell Lung[Title/Abstract]) OR Non-Small Cell Lung Cancer[Title/Abstract]) OR NSCLC[Title/Abstract] | 66505 |
| #3: | #1 OR #2 | 75042 |
| #4: | Search "Nivolumab"[Mesh] | 2152 |
| #5: | Search (((((Nivolumab[Title/Abstract]) OR Pembrolizumab[Title/Abstract]) OR Atezolizumab[Title/Abstract]) OR Avelumab[Title/Abstract]) OR Durvalumab[Title/Abstract]) OR Cemiplimab[Title/Abstract] | 6646 |
| #6: | #4 OR #5 | 7093 |
| #7: | Search ((randomized controlled trial[Publication Type] OR (randomized[Title/Abstract]) | 756649 |
| #8: | #3 AND #6 AND #7 | 184 |


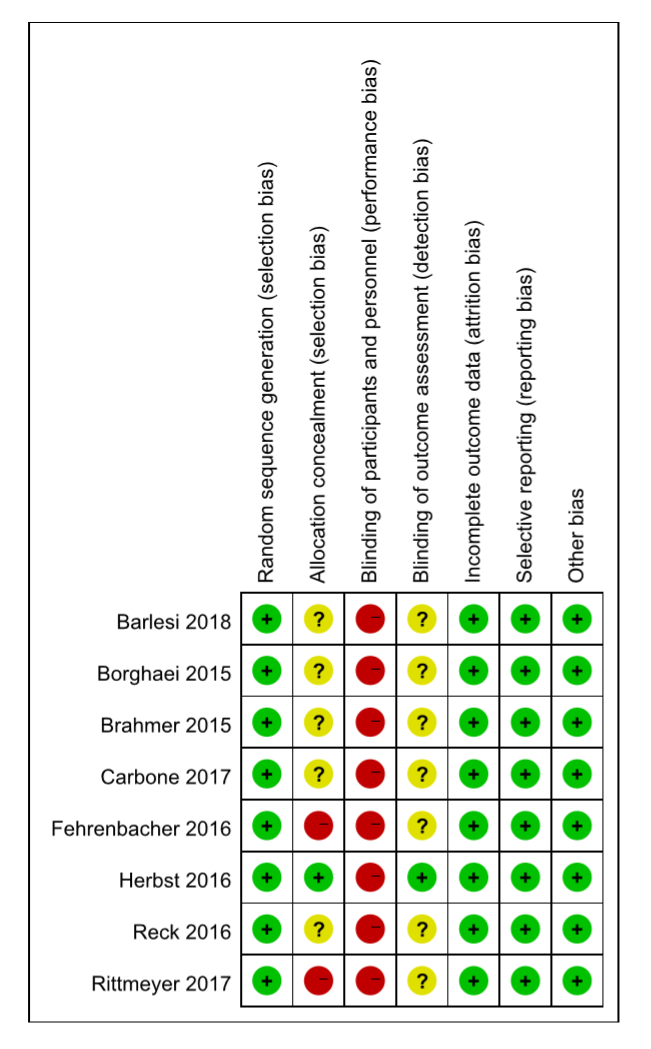


**Supplementary FIGURE 1** Risk of bias graph for RCTs based on Cochrane risk assessment tool
